# Comparison of Energy Expenditure Observed between Scheduled Activities in Collegiate Team-Sport Female Athletes

**DOI:** 10.3390/sports9040050

**Published:** 2021-04-02

**Authors:** Jessica M. Moon, Hannah A. Zabriskie, Patrick S. Harty, Bradley S. Currier, Julia C. Blumkaitis, Richard A. Stecker, Andrew Jagim, Chad M. Kerksick

**Affiliations:** 1 Exercise and Performance Nutrition Laboratory, School of Health Sciences, Lindenwood University, 209 S. Kingshighway, St. Charles, MO 63301, USA; jmm805@lindenwood.edu (J.M.M.); currierb@mcmaster.ca (B.S.C.); julia.blumkaitis@sbg.ac.at (J.C.B.); rstecker@lindenwood.edu (R.A.S.); 2 Department of Kinesiology, Towson University, Towson, MD 21252, USA; hzabriskie@towson.edu; 3 Energy Balance and Body Composition Laboratory, Department of Kinesiology & Sport Management, Texas Tech University, Lubbock, TX 79409, USA; Patrick.Harty@ttu.edu; 4 Sports Medicine, Mayo Clinic Health System, Onalaska, WI 54650, USA; jagim.andrew@mayo.edu

**Keywords:** energy balance, women, sports, basketball, lacrosse, body composition

## Abstract

Energy needs of female team-sport athletes are poorly understood with no evidence highlighting differences present between scheduled activities. The purpose of this study was to examine the difference in energy expenditure between NCAA Division II female basketball (BBALL) and lacrosse (LAX) athletes during different scheduled team activities. Female BBALL (*n* = 13; 19.8 ± 1.3 yrs; 173.9 ± 13.6 cm; 74.6 ± 9.1kg; 27.1 ± 3.2%fat) and LAX (*n* = 20; 20.4 ± 1.8yrs; 168.4 ± 6.6cm; 68.8 ± 8.9kg; 27.9 ± 3.1%fat) athletes were outfitted with heart rate and activity monitors during four consecutive days on five different occasions (20 days total) across an entire academic year to assess differences in total daily activity energy expenditure (TDEE), activity energy expenditure (AEE), and physical activity level (PAL). Data were categorized by type of scheduled daily activities: Practice, Game, Conditioning, or Off. Independent of day type, TDEE, AEE, and PAL levels were greater (*p* < 0.05) in BBALL athletes. For each sport, TDEE, AEE, and PAL were significantly different (*p* < 0.05) between classified activity days. BBALL and LAX athletes experienced higher values on game days for TDEE, AEE, and PAL, with the lowest values experienced on off days. In conclusion, calculated levels of TDEE, AEE, and PAL in female collegiate BBALL and LAX athletes were determined to be different, irrespective of the scheduled activity.

## 1. Introduction

Human physiology requires a constant supply of energy to sustain life and support physiological functions. The total amount of energy expended each day is referred to as total daily energy expenditure (TDEE) and consists of activity energy expenditure (AEE), resting metabolic rate (RMR), thermic effect of food (TEF), and non-exercise activity thermogenesis (NEAT). RMR is a consistent and primary contributor to TDEE comprising nearly 60–65% of TDEE, whereas AEE can vary widely between individuals and within the same person [1,2]. Individuals who expend significant amounts of energy, such as competitive athletes, must be conscious of their energy requirements to allow for adequate matching of their expended energy with dietary intake to maintain a state of energy balance. Regulation of energy balance is a primary focus for athletes looking to promote effective training, stave off illness, achieve desired training adaptations, and facilitate recovery both completely and efficiently [3]. When calories are consumed in excess of TDEE, weight gain and changes in body composition often occur. Conversely, insufficient calorie consumption places individuals at risk of being in a negative energy balance or low energy availability (the amount of energy available to devote to physiological tasks). Prolonged periods of low energy availability or negative energy balance is considered by many sport scientists to be the hallmark characteristic of relative energy deficiency in sport (RED-S), which is associated with an increased risk for injury, overtraining syndrome, decreased performance, reduced training adaptations, longer recovery times, and compromised immune systems [4,5,6,7,8,9,10]. If low energy availability persists, an increased likelihood of reduced bone mineral density, menstrual cycle dysfunction, weight loss, or other physiological states of dysfunction can occur [9,11,12,13]. For these reasons, energy availability measurements as well as internal and external load monitoring have begun to garner more interest from athletic populations and athletic support staff.

In this respect, the available literature is limited primarily to studies that have examined the energy status of athletes across entire training seasons and the majority of this research has focused on male athletes [14,15,16,17,18]. Assessments of the energy status of female athletes in team sports [16,19,20,21,22,23,24] as well as endurance athletes [13,25,26,27] are available, while two recent studies monitored collegiate (NCAA Division II) female lacrosse [28], and basketball [29] athletes for changes in energy turnover that occurred throughout different parts of the season. Both groups of athletes exhibited the highest levels of TDEE during the pre-season in comparison to values derived during the season. Additionally, the lacrosse and basketball athletes displayed the highest levels of activity energy expenditure (AEE) during the pre-season and in-season data collection windows while the lowest levels occurred during the post-season or off-season collection windows. From this data, two important issues persist, particularly as they relate to female athletes. First, no between-sport comparisons are present. This point is extremely important as it helps to demonstrate and emphasize the individual considerations that may be needed relative to fueling needs, budget for food, and access to food and fueling while competing and travelling. Second, no studies to date, have provided insight into the magnitude of change observed in energy turnover relative to the type of team activities being completed on any given day. It is well accepted that changes in energy requirements of athletes vary based on sport type, position, level of competition, competition schedules, and seasonal training demands with further considerations being needed for athletes who are required to compete on a frequent basis or travel for competition [17,18,30,31]. Collectively, all of these factors combine to make it challenging for an athlete, sport coach, strength and conditioning coach, researcher, dietitian, or nutritionist to understand the magnitude of changes that can occur in energy expenditure relative to the type of activities occurring on any given day, in addition to the magnitude of differences between sports. It has been clearly shown in previous studies that athletes and sport coaches require additional education and training regarding proper strategies to improve athlete health and performance [32,33,34,35,36]. An increased understanding of how energy needs are different between an off day and a game day or between an off day and a conditioning or practice day will help reinforce the importance of manipulating energy intake to best match the training that will or has occurred as part of a nutritional periodization plan. Alternatively, this also means a coach can manipulate the prescribed training load to facilitate better matching of fueling needs to optimize performance and recovery. As concepts of nutritional periodization continue to grow in popularity [37,38], it is important for athletes and coaches to extend these approaches to include a more personalized management of energy turnover.

While this information will be helpful to guide decisions for coaches, trainers, and dietitians involving the entire team, this information will be particularly helpful for those athletes who need to modify their body composition to optimize their performance potential. A better understanding of the magnitude of energy turnover from one day to the next will provide a valuable foundation of information when educating an athlete who struggles with understanding the importance of fueling their body for optimal health, performance, and recovery. For these reasons, the purpose of the present study was to examine the differences in TDEE, AEE, and physical activity levels (PAL) between four different types of scheduled team activities: Off Days, Practice Days, Conditioning Days, and Game days in two female team sports.

## 2. Methods

### 2.1. Experimental Design

This observational study builds upon previously published data from two separate studies which spanned an entire academic year (i.e., off-season, pre-season, and competitive season) for women’s collegiate basketball [29] and lacrosse [28]. The current sample consists of all complete participant data sets from the two separate trials. Briefly, data collection for the studies began during the beginning of the fall academic semester and concluded during the spring academic semester encompassing five different phases of data collection throughout the 2016–2017 academic year for basketball players, and during the 2017–2018 academic year for lacrosse players. During each phase, participants were monitored over four consecutive days (two weekend days, two weekdays) using a combined heart rate and accelerometer (Actiheart, CamNTech, Cambridge, UK) for a total of 20 monitored days. Data specific to AEE, TDEE, PAL, type of scheduled team activity, and body composition were collected. Each day of monitoring was subsequently classified by researchers and coaches into one of four types of days: (1) Off—no team related activity or training was completed; (2) Practice—a scheduled team sport-specific skills practice took place; (3) Conditioning—subjects participated in a scheduled team conditioning and/or monitored lifting session, and (4) Game—an officially sanctioned game was completed. On days when a team sport-specific skill practice and a scheduled team lifting session occurred, the day was classified as a practice day.

### 2.2. Subjects

A total of 33 female NCAA Division II team sport athletes were assessed. The entire cohort consisted of 13 basketball (19.8 ± 1.3 yrs., 173.9 ± 13.6 cm, 74.6 ± 9.1 kg, 27.1 ± 3.2% body fat) and 20 lacrosse athletes (20.4 ± 1.8 yrs., 168.4 ± 6.6 cm, 68.8 ± 8.9 kg, 27.9 ± 3% body fat). Athletes were recruited through their respective teams during a team meeting where all study details, participation requirements, and study designs were outlined. Inclusion criteria consisted of those athletes that were members of the team upon which we collected the data. Each athlete was required to have been medically cleared by their team physician prior to the start of their athletic seasons before their respective team’s initial observational study. All athletes who suffered some form of musculoskeletal injury that limited their ability to fully participate in team activity for more than two weeks were excluded. Written informed consent was provided and all procedures were approved by Lindenwood University’s Institutional Review Board (Lacrosse: Protocol # IRB-19-L0012, Approved: 25 August 2017; Basketball: Protocol # 964572, Approved: 16 October 2016).

### 2.3. Procedures

#### 2.3.1. Anthropometrics and Body Composition

Body mass, height, and body composition were measured before any energy expenditure data being collected at the beginning of the academic year. Shoes and excess clothing were removed followed by body mass being obtained to the nearest 0.1 kg (Digital Scale BWB627A Class III, Tanita, Tokyo, Japan). Height was measured to the nearest centimeter using a wall-mounted stadiometer (HR-200, Tanita, Tokyo, Japan). Body composition was obtained using dual-energy X-ray absorptiometry (DEXA) for basic demographic information and for normalization of collected energy data. To standardize testing conditions before completion of the DEXA, participants were instructed to arrive at the laboratory after observing an overnight 8–10 h fast and avoiding exercise for at least 24 h before testing. Calibration procedures were completed before testing and all scans were completed using a Hologic QDR Discovery A (HOLOGIC, Bedford, MA, USA) and analyzed using the accompanying software (Hologic APEX Software, Version 4.5.3; HOLOGIC) to obtain body composition and bone density parameters. Test–retest reliability (ICC and CV) using these procedures in 40 healthy college-aged men and women was determined to be 0.99% and 1.26% for DEXA fat and 0.99% and 0.75% for DEXA fat-free mass (data not shown). All results were calculated using the NHANES correction factor.

#### 2.3.2. Physical Activity Monitoring

Physical activity monitoring occurred over the course of 20 days throughout five separate points in the competitive season. During each measurement phase, energy expenditure data were collected for a total of four days with monitoring occurring consecutively on two weekdays and two weekend days. Each subject was outfitted with a physical activity monitor (Acti-Heart; CamNTech, Inc., Boerne, TX, USA) that was integrated with heart rate and uniaxial accelerometers. Athletes were instructed to wear the monitor at all times with the exception of showering. As perspiration sometimes inhibited the adhesion of the electrodes, during activity, LAX athletes were provided with a removable chest strap to which the monitor connected. This option was not made available to BBALL athletes. The monitor was attached using two standard ECG electrodes with one being positioned over the xiphoid process and the other on the left side of the chest below the left breast and between the fifth and sixth rib. The placement of the monitors was determined by manufacturer recommendations with the lead wire being placed parallel to the ground. Monitors were initially placed by a study team member and the location of the electrode was circled with a permanent marker so that application of new electrodes could be replicated by subjects as needed. Heart rate was continuously collected every minute for the entire data collection period and was combined with accelerometry data to calculate AEE. All measures: AEE, TDEE, and PAL, were determined using Acti-Heart software as it has previously been shown be a valid measure for the calculation of AEE and TDEE in adults [39].

#### 2.3.3. Day Classification

Team activities were determined by coaches and researchers during each monitoring period and classified into one of the following categories: Off, Practice, Conditioning, or Game days. Off days consisted of monitored periods where no team related physical activity or training was performed. Practice days consisted of a period of monitoring where the subject participated in a scheduled sport-specific skills practice. Conditioning days consisted of participation in a scheduled team conditioning and/or monitored lifting session. Game days comprised monitored days when subjects competed in a scheduled team game. In the case that a practice occurred on the same day as a team lift, the day was categorized as Practice.

### 2.4. Statistical Analysis

All statistical analysis was completed in JASP Team (JASP (Version 0.14)). All data are presented as means ± standard deviation. All data were checked for normality and assumptions before analysis. Between sport differences for raw data and data normalized to body mass, lean mass, and fat-free mass, for TDEE, AEE and PAL regardless of day type, were assessed using a repeated measures ANOVA. Within each sport, a repeated measures ANOVA was used to identify differences between the types of days for both raw data and data normalized to body mass, lean mass, and fat-free mass. A 2 × 4 (sport × type of day) mixed factorial ANOVA with repeated measures on time was used to calculate main effects for time and sport as well as the interaction effect between sport and type of day. When sport × type of day interaction effects were present, paired sample *t*-Tests were used as post hoc comparisons. A significance level of α = 0.05 was used.

## 3. Results 

### 3.1. Participant Anthropometrics by Sport

A total of 33 female NCAA Division II team sport athletes completed all assessments (Table 1). Between-sport differences were identified for height (*p* = 0.004), lean mass (*p* = 0.003) and fat-free mass *p* = 0.002) with BBALL athletes being significantly taller and having greater levels of lean and fat-free mass, respectively.

### 3.2. Total Daily Energy Expenditure

**Figure 1:**
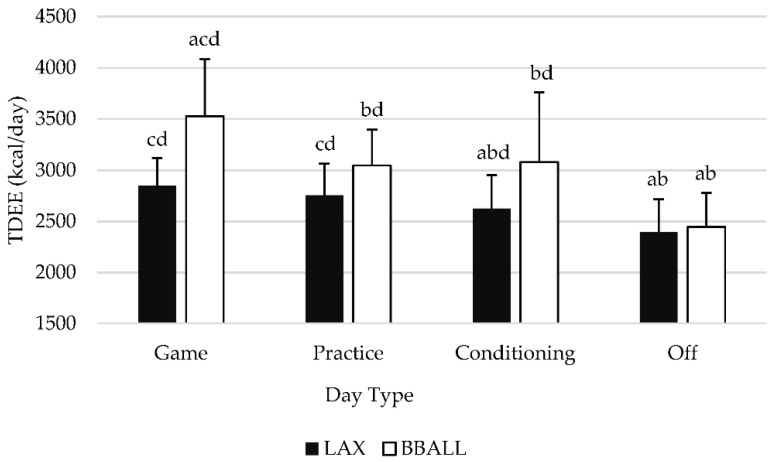
Differences in total daily energy expenditure (TDEE, kcals/day) categorized by type of activity completed. Means with different letters are significantly different than one another (*p* < 0.05). a is significantly different from practice; b is significantly different from game; c is significantly different from conditioning; d is significantly different from off. Data is presented as means ± SD.

A significant main effect was observed (*p* = 0.002) between sports regardless of type of day with basketball athletes having a significantly higher TDEE than lacrosse athletes. When normalized to body mass, lean mass and fat free mass, no significant effects were observed. When day type was examined individually for each sport (Figure 1), BBALL athletes observed significant differences (*p* < 0.05) between all types of days with the exception of conditioning and practice days (*p*= 0.934). Specifically, TDEE was significantly higher on game days, compared to practice (*p* = <0.001), conditioning (*p* = 0.020) and off (*p* < 0.001) days, while off days were significantly lower than all other days. For LAX athletes, a significant difference was observed between game, conditioning and off days with game days producing significantly higher TDEE values than conditioning (*p* = 0.003) and off (*p* < 0.001) days. Practice days were significantly higher than both conditioning (*p* = 0.004) and off (*p* < 0.001) days, while off days produced significantly lower (*p* < 0.001) TDEE values than every other day.

Table 2 provides a summary of all raw data after being normalized to body mass (BM), lean mass (LM), and fat-free mass (FFM) (Table 2). When normalized to BM, LM, and FFM, BBALL athletes exhibited significant differences between all days (*p* < 0.05) with the exception of conditioning and practice days (BM: *p* = 0.779, LM: *p* = 0.888, FM: *p* = 0.888). Additionally, TDEE was significantly higher on game days, compared to practice (BM: *p* < 0.001, LM; *p* < 0.001, FFM: *p* < 0.001), conditioning (BM: *p* = 0.007, LM: *p* = 0.006, FFM: *p* = 0.006), and off days (BM: *p* < 0.001, LM: *p* < 0.001, FFM: *p* < 0.001) days, with off days being significantly lower than all other days. For LAX athletes, a significant difference was observed between all days (*p* < 0.05) with game days producing significantly higher TDEE values than conditioning (BM: *p* = 0.001, LM: *p* = 0.002, FFM: *p* = 0.002), practice (BM: *p* = 0.035, LM: *p* = 0.039, FM: *p* = 0.040), and off days (BM: *p* < 0.001, LM: *p* < 0.001, FFM: *p* < 0.001). Practice days were significantly higher than both conditioning (BM: *p* = 0.003, LM: *p* = 0.003, FFM: *p* = 0.003) and off days (BM: *p* < 0.001, LM: *p* < 0.001, FFM: *p* < 0.001), while off days produced significantly lower TDEE values (BM: *p* < 0.001, LM: *p* < 0.001, FFM: *p* < 0.001) than every other day.

### 3.3. Activity Energy Expenditure

**Figure 2:**
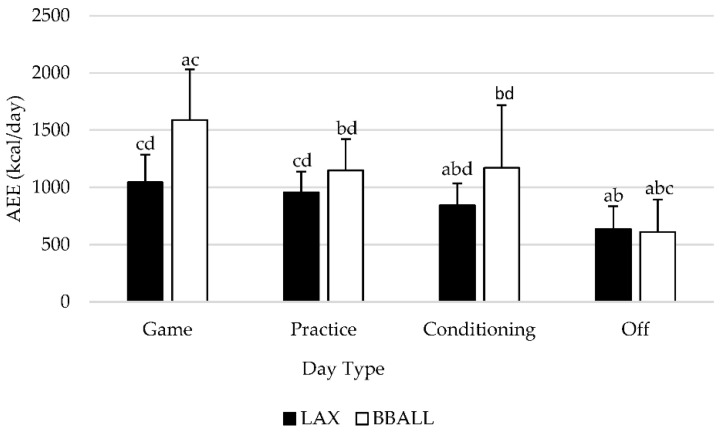
Differences in activity energy expenditure (AEE, kcals/day) categorized by type of activity completed. Means with different letters are significantly different than one another (*p* < 0.05). a is significantly different from practice; b is significantly different from game; c is significantly different from conditioning; d is significantly different from off. Data is presented as means ± SD.

A significant main effect was observed (*p* = 0.001) between sports regardless of type of day with basketball athletes having a significantly higher AEE than lacrosse athletes. When examined by individual sport and day type (Figure 2), a significant difference (*p* < 0.05) was observed for BBALL athletes for all days with the exception of conditioning and practice days (*p* = 0.934). AEE was significantly higher on game days, compared to practice (*p* < 0.001), conditioning (*p* = 0.018) and off days (*p* < 0.001), with off days being significantly lower than all other days. For LAX athletes, a significant difference was observed between game, conditioning and off days with game days producing significantly higher AEE values than conditioning (*p* = 0.003) and off (*p* < 0.001) days. No significant difference was observed between game and practice days (*p* = 0.091). Practice days were significantly higher than both conditioning (*p* = 0.004) and off (*p* < 0.001) days, while off days produced significantly lower (*p* < 0.001) AEE values when compared to all other day categories.

As seen in Table 2, raw AEE data were normalized to BM, LM, and FFM and compared between types of days. Using normalized data, BBALL athletes experienced a significant difference (*p* < 0.05) between all days with the exception of conditioning and practice days (BM: *p* = 0.845, LM: *p* = 0.922, FFM: *p* = 0.922). AEE was significantly higher on game days, compared to practice (BM: *p* < 0.001, LM: *p* < 0.001, FFM: *p* < 0.001), conditioning (BM: *p* = 0.006, LM: *p* = 0.005, FFM: *p* = 0.005), and off days (BM: *p* < 0.001, LM: *p* < 0.001, FM: *p* < 0.001), with off days being significantly lower than all other days. For LAX athletes, a significant difference was observed between all days (*p* < 0.05) with game days producing significantly higher AEE values than conditioning (BM: *p* = 0.001, LM: *p* = 0.002, FFM: *p* = 0.002), practice (BM: *p* = 0.029, LM: *p* = 0.033, FFM: *p* = 0.034), and off days (BM: *p* < 0.001, LM: *p* < 0.001, FFM: *p* < 0.001). Practice days were significantly higher than both conditioning (BM: *p* = 0.003, LM: *p* = 0.003, FFM: *p* = 0.003) and off days (BM: *p* < 0.001, LM: *p* < 0.001, FFM: *p* < 0.001), while off days produced significantly lower AEE values (BM: *p* < 0.001, LM: *p* < 0.001, FFM: *p* < 0.001) than every other type of day.

### 3.4. Physical Activity Levels

**Figure 3:**
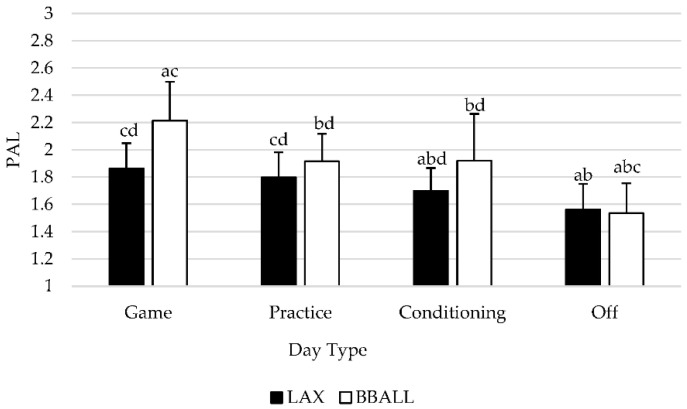
Differences in physical activity level (PAL) categorized by type of activity completed. Means with different letters are significantly different than one another (*p* < 0.05). a is significantly different from practice; b is significantly different from game; c is significantly different from conditioning; d is significantly different from off. Data is presented as means ± SD.

A significant main effect was observed (*p* = 0.010) between sports regardless of type of day with BBALL athletes having a significantly higher PAL than LAX athletes. When examined by individual sport and day type (Figure 3), significant differences (*p* < 0.05) were observed for BBALL athletes for all days with the exception of conditioning and practice days (*p* = 0.880). PAL was significantly higher on game days, compared to practice (*p* < 0.001), conditioning (*p* = 0.023) and off days (*p* < 0.001), with off days being significantly lower than game (*p* < 0.001), practice (*p* < 0.001) and conditioning (*p* = 0.022) days. For LAX athletes, a significant difference was observed between game, conditioning and off days with game days producing significantly higher PAL values than conditioning (*p* = 0.002) and off (*p* < 0.001) days. Practice days were significantly higher than both conditioning (*p* = 0.003) and off (*p* < 0.001) days, while off days produced significantly lower (*p* < 0.001) PAL values than every other day.

## 4. Discussion

The purpose of this investigation was to assess the differences between scheduled team activities regarding TDEE, AEE, and PAL in NCAA Division II female lacrosse and basketball athletes. When examined by type of day, basketball athletes experienced significant differences in TDEE, AEE, and PAL between all day types, with game days producing significantly higher values than all other days. Practice and conditioning days values did not differ from each other, while off days were lower than every other scheduled activity. When normalized to BM, LM, and FFM, significant differences for BBALL athletes did not deviate from the pattern of absolute values. For LAX athletes, significant differences for absolute values were seen between multiple day types for TDEE, AEE and PAL with game days producing significantly higher values than conditioning and off days. Game and practice days did not significantly differ and produced similar responses while off days were significantly lower than all other active days. When values were normalized to BM, LM, and FFM, significant differences were detected between all day types including game and practice days. Between-sport differences were identified for height, LM, and FFM, with basketball athletes being significantly taller and having greater levels of LM and FFM, respectively, compared to lacrosse athletes. Regardless of type of day and if data were represented in absolute terms or normalized to body mass, lean mass, or fat-free mass, basketball athletes had significantly higher TDEE, AEE, and PAL compared to lacrosse athletes.

Recently more reports have become available that provide information on energy turnover within team sports [17,22,28,29,30,40]. These reports document various athlete populations throughout various phases of their athletic competition in efforts to examine the large negative energy balances that routinely occur throughout their competitive seasons [28,29,41]. However, the extent to which various energy components differ between different scheduled activities has been examined lacks adequate information. Briggs et al. [40] evaluated energy intake and expenditure in ten professional English Premier League club adolescent male soccer players was measured across a single competitive week with days being categorized into heavy training days, moderate training days, rest days, and a match day (4 training days, 2 rest days, and 1 match day). In agreement with our findings for both lacrosse and basketball athletes, match and heavy training days revealed significantly higher EE values compared to rest days. Similarly, Mara et al. [22] conducted an analysis of energy expenditure of elite female soccer players across a training week and subsequently reported that game days resulted in the highest levels of energy expenditure, followed by training days and finally rest days. In a similar study conducted by Tooley et al. [42], ten male Professional Rugby League athletes were observed over four training days a pre-match day, a match day, and two non-training recovery days. It was found that energy intake remains relatively stable across the week, despite changing energy expenditure values depending on the intensity of the training. Although Tooley et al. [42] categorized the athletes’ activity days, statistical comparisons between: match vs. recovery, match vs. training, and training vs. recovery days were not presented. Instead, activities were reported to calculate appropriate energy expenditures based on activity to analyze energy balances within the athletes across the entire week. The previously described studies by Briggs et al. [40], Mara et al. [22] and Tooley et al. [42], suggest that players’ energy intakes may need to be adjusted for the type of training day to account for the observed changes in training intensity, and volume to maintain energy balance. The present study supports these findings as significantly higher TDEE, AEE, and PAL levels were observed for both lacrosse and basketball athletes on days where different activities and levels of intensity were performed.

Data from these female collegiate basketball [29] and lacrosse [28] cohorts have been previously published, focusing on the observed changes in body composition, dietary intake, and energy status indicators throughout each sport’s respective season. Energy expenditure data, however, were not examined as it relates to specific activities being performed during a given day. When examined across an entire collegiate season, basketball athletes were found to have lower TDEE and PAL values as the season progressed; however, a negative energy balance was still observed across all time points in the season [29]. Similarly, lacrosse athletes were found to have significant differences in TDEE throughout their season with pre-season training resulting in the highest energy expenditures. Additionally, negative energy balance and low energy availability values were also reported at all measurement points [28]. To our knowledge no other study has assessed TDEE, AEE, and PAL as they relate to different activity types across different sports. Results from the present study provide novel insight into the energetic requirements of specific behavioral activities that athletes regularly participate in and provide further details why periodized energy value and macronutrient intakes may be needed to best match anticipated changes in energy expenditure. Periodized nutritional intake may be advised to account for changes in training volumes and intensities [37] in order to reflect adequate energy intake with specific types of training days. By either decreasing or increasing caloric consumption to avoid negative energy balances on a day by day basis, performance, growth, and development can be optimized [43,44] while also reducing risk of low energy availability and RED-s.

These data have a few limitations for the reader to consider. The primary limitation stemmed from our lack of confirmatory physical activity data via logs or questionnaires to confirm physical activity. Athletes behaviors outside of team-directed activities may have included self-directed exercise, recreational game play, etc., that were captured by activity monitors ultimately contributing to TDEE but not included as part of our defined days. Thus, the administration of daily physical activity logs would have aided in the validation of TDEE and AEE measurements and is recommended for future research in this area. Additionally, some degree of over-estimation in our data may have confounded the accuracy of our outcomes. In this respect, other studies [45] have indicated an over-estimation of energy expenditure in female adolescent runners can occur when using the combined heart and accelerometer monitors used in this study. Notably, the length of time where the monitors were used by Nichols et al. [45] in comparison to the present study was less frequent and shorter in duration than in the present investigation. Additionally, expenditure data from the lacrosse athletes was analyzed using an accelerometer only model while the basketball athletes used a combination of heart rate and accelerometry data. The monitors have multiple models upon which analysis can be completed and as a result, some aspect of the observed differences between the two groups of athletes could be explained by the differences in the analytical models that were employed.

In conclusion, an investigation between two groups of collegiate NCAA Division II female athletes with specific regard to different scheduled team activities indicated that energy expenditure was significantly higher on game days than all other days for both athletic cohorts, while practice and conditioning days produced mixed significance dependent on each sport. Additionally, off days produced significantly lower energy expenditure values than all other days regardless of sport type. Further, irrespective of the type of scheduled team activities and how the data were represented, basketball athletes reported higher levels of energy expenditure than lacrosse athletes. Finally, normalization of all energy data to body mass, lean mass, or fat-free mass did not change any of the observed differences.

## Figures and Tables

**Table 1:** Anthropometric information by type of sport.

Variable	Basketball (*n* = 13)	Lacrosse (*n* = 20)	*p*-Value
Age (years)	19.8 ± 1.3	20.4 ± 1.8	0.061
Height (cm)	173.9 ± 13.6	168.4 ± 6.6	0.004 ^†^
Weight (kg)	74.6 ± 9.1	68.8 ± 8.9	0.097
DEXA Body Fat %	27.1 ± 3.2	27.9 ± 3.1	0.847
DEXA Lean Mass (kg)	51.7 ± 6.9	44.5 ± 5.1	0.003 ^†^
DEXA Fat-Free Mass (kg)	54.7 ± 7.1	47.0 ± 5.3	0.002 ^†^

Data is presented as means ± SD. *p*-value is from independent *t*-test between sport types for each variable. ^†^ Significantly different between sports (*p* < 0.05). DEXA = Dual-energy X-ray absorptiometry.

**Table 2:** Differences in total daily energy expenditure (TDEE) and activity energy expenditure (AEE) normalized to body mass, lean mass, and fat-free mass.

Variable	Type of Day	Basketball	Lacrosse
TDEE/kg body mass	Practice	41.4 ± 5.7 ^b,d^	40.2 ± 3.3 ^b,c,d^
	Game	47.7 ± 6.8 ^a,c,d^	41.8 ± 5.2 ^a,c,d^
	Conditioning	41.2 ± 6.4 ^b,d^	38.2 ± 2.5 ^a,b,d^
	Off	33.3 ± 6.2 ^a,b,c^	34.9 ± 3.2 ^a,b,c^
	All Days	40.8 ± 5.2	40.8 ± 3.1
TDEE/kg lean mas	Practice	60.1 ± 8.0 ^b,d^	62.1 ± 3.9 ^b,c,d^
	Game	69.3 ± 10.1 ^a,c,d^	64.6 ± 7.3 ^a,c,d^
	Conditioning	60.0 ± 8.0 ^b,d^	59.1 ± 4.1 ^a,b,d^
	Off	48.3 ± 8.8 ^a,b,c^	53.8 ± 3.7 ^a,b,c^
	All Days	59.3 ± 8.0	59.9 ± 3.9
TDEE/kg FFM	Practice	56.8 ± 7.4 ^b,d^	58.6 ± 3.6 ^b,c,d^
	Game	65.5 ± 9.4 ^a,c,d^	61.0 ± 6.8 ^a,c,d^
	Conditioning	56.7 ± 10.3 ^b,d^	55.8 ± 3.7 ^a,b,d^
	Off	45.7 ± 8.2 ^a,b,c^	50.9 ± 3.4 ^a,b,c^
	All Days	56.0 ± 7.4	56.6 ± 3.5
AEE/kg body mass	Practice	15.7 ± 4.4 ^b,d^	14.0 ± 2.5 ^b,c,d^
	Game	21.5 ± 5.6 ^a,c,d^	15.5 ± 4.1 ^a,c,d^
	Conditioning	15.6 ± 5.6 ^b,d^	12.2 ± 2.2 ^a,b,d^
	Off	9.0 ± 4.9 ^a,b,c^	9.2 ± 2.7 ^a,b,c^
	All Days	15.1 ± 4.1 ^†^	12.7 ± 2.3 ^†^
AEE/kg lean mass	Practice	22.7 ± 6.3 ^b,d^	21.5 ± 3.2 ^b,c,d^
	Game	31.2 ± 8.4 ^a,c,d^	23.8 ± 6.0 ^a,c,d^
	Conditioning	22.8 ± 8.9 ^b,d^	18.9 ± 3.3 ^a,b,d^
	Off	12.2 ± 6.8 ^a,b,c^	14.2 ± 3.6 ^a,b,c^
	All Days	22.1 ± 6.1	19.6 ± 3.1
AEE/kg FFM	Practice	21.5 ± 5.8 ^b,d^	20.3 ± 3.0 ^b,c,d^
	Game	29.5 ± 7.8 ^a,c,d^	22.5 ± 5.7 ^a,c,d^
	Conditioning	21.5 ± 8.4 ^b,d^	17.8 ± 3.1 ^a,b,d^
	Off	11.6 ± 6.4 ^a,b,c^	13.4 ± 3.4 ^a,b,c^
	All Days	20.9 ± 5.7	18.5 ± 2.9

^†^ Significantly different between sports (*p* < 0.05). Means with different letters are significantly different than one another (*p* < 0.05). ^a^ is significantly different from practice; ^b^ is significantly different from game; ^c^ is significantly different from conditioning; ^d^ is significantly different from off. All units of measure are in kcals normalized to kg of body tissue. Data is presented as means ± SD.

## Data Availability

The data presented in this study are available on request from the corresponding author.
